# Culture and characterization of oral mucosal epithelial cells on human amniotic membrane for ocular surface reconstruction

**Published:** 2008-01-30

**Authors:** Soundarya Lakshmi Madhira, Geeta Vemuganti, Anirban Bhaduri, Subhash Gaddipati, Virender Singh Sangwan, Yashoda Ghanekar

**Affiliations:** 1 Sudhakar and Sreekanth Ravi Stem Cell Biology Laboratory; 2 Ophthalmic Pathology Services; 3 Ophthalmic Plastics, Orbit and Ocular Oncology Services; 4 Cornea and Anterior Segment Services, L.V. Prasad Eye Institute, Hyderabad, India

## Abstract

**Purpose:**

To culture oral mucosal epithelial cells on deepithelialized human amniotic membrane without the use of feeder cells and to compare the characteristics of cultured oral cells with cultured limbal and conjunctival epithelial cells for use in ocular surface reconstruction.

**Methods:**

Oral biopsies were obtained from healthy volunteers after informed consent and were cultured on deepithelialized amniotic membrane for three to four weeks. Confluent cultures of limbal, oral, and conjunctival cells were subjected to characterization of markers of stem cells and of epithelial differentiation by reverse-transcription polymerase chain reaction (RT–PCR) and by immunohistochemistry. Ultrastructural studies were also performed using electron microscopy.

**Results:**

A sheet of healthy, stratified oral epithelial cells was obtained within three to four weeks of culture. Electron microscopy demonstrated that the cells formed gap junctions and desmosomes. RT–PCR analysis showed that cultured oral epithelial cells expressed markers of epithelial differentiation such as cytokeratin K3, K4, K13, K15 and connexin 43. The cells also expressed stem cell markers of epithelial cells such as ΔN isoforms of p63 as well as p75, a marker for stem cells of oral epithelium. The cells did not express cytokeratin K12 or Pax-6, an eye-specific transcription factor.

**Conclusions:**

Oral epithelial cells can be cultured as explants on deepithelialized amniotic membrane without using feeder cells. Characterization showed that these cells maintain the phenotypic characteristics of oral epithelial cells and that the culture is a heterogeneous population of differentiated cells and stem cells. We find the cultured oral epithelial cells usable for ocular surface reconstruction in patients having bilateral ocular surface diseases.

## Introduction

A loss of limbal stem cells due to injury or systemic illness leads to severe ocular surface disorders that can result in the loss of vision and severe discomfort. As these stem cells surrounding the cornea are responsible for the regeneration of the corneal epithelium [1,2], any condition causing the loss of these stem cells or of the limbal niche leads to the loss of the corneal epithelium and to severe ocular surface disease. Chemical or thermal injuries, ultraviolet (UV) and ionizing radiations, severe microbial infection, surgeries and cryotherapy of the limbal region, or conditions like Stevens-Johnson syndrome and aniridia can lead to limbal stem cell deficiency (LSCD). LSCD results in dryness, discomfort, conjunctivalization of the ocular surface and neovascularization, which leads to corneal opacity, and finally to blindness [3-5]. LSCD can be treated by transplantation of the limbal tissue from a healthy eye either by direct transfer [6] or after cultivating it in vitro on a suitable matrix such as the amniotic membrane [7-10]. We have used this technique to treat over 500 patients with a success rate of 70% [11].

However, bilateral LSCD requires allogenous limbal tissue as a source of limbal stem cells, and this necessitates long term use of systemic immunosuppressants to avoid graft rejection [12,13]. Immunosuppressants have several side effects that affect the quality of the patient’s life and are expensive. There is also the risk of rejection and graft failure despite immunosuppression. Therefore, sources of autologous tissue that can functionally replace the corneal epithelium have been considered as an alternative to allogenous limbal transplants.

Since the corneal epithelium is of the stratified squamous type, autologous epithelial cells such as oral, conjunctival, nasal, esophageal, rectal, and vaginal epithelia, which all have a similar morphology, could be considered as an alternative to allogenous limbal transplants. The potential of conjunctival epithelium has been explored by some investigators as an alternative to cultured limbal cells, and these studies suggest that transplantation of cultured conjunctival cells is a better option than amniotic membrane graft alone if transplantation of cultured limbal cells is not possible [14,15]. More extensive studies have been performed to check the feasibility of using oral mucosal epithelium for this purpose as it is easily available and can be harvested without invasive surgery. These studies suggest that oral mucosal epithelium is a feasible alternative for allogenous limbal transplants [16-21]. Oral mucosal epithelium cultured on the human amniotic membrane with the help of feeder cells has been characterized extensively, and has been used to reconstruct the ocular surface in rabbits [16] as well as humans with chemical injury and Stevens-Johnson syndrome [17]. In the longest study reported so far, cultured oral mucosal epithelial cells were transplanted in patients with LSCD and followed up for up to 34 months [18]. The results from this study are promising, showing formation of a stable ocular surface in patients and improvement in visual acuity. Oral epithelial cells have also been cultured on a temperature-responsive cell culture surface with [19,20] or without [21] NIH/3T3 feeder layers and used for ocular surface reconstruction.

Here, we report an improved method for the culture of oral epithelial cells on deepithelialized amniotic membrane using an explant culture technique without the use of any feeder cells. To check the feasibility of using these cells as an alternative to limbal cells, we have compared the characteristics of these cultured cells with those of cultured limbal cells. In addition, we have also investigated the characteristics of conjunctival cells cultured in a similar way to check if it can be used for ocular surface reconstruction.

## Methods

### Harvesting of limbal, conjunctival, and oral mucosal epithelial tissues

Studies were performed after the protocol was approved by the Institutional Review Board. Experiments were performed using oral tissue harvested from healthy adult volunteers (age 18–60 years) after obtaining informed consent and performing a pre-surgical evaluation of ocular and oral health. Volunteers with a history of smoking, chewing tobacco, and oral infection/inflammation were excluded. Twenty-five oral biopsies were used for this study. Oral hygiene was optimized with preoperative 1% Betadine mouthwash for three days. A mucosal biopsy of 3 mm×3 mm was harvested from the buccal surface of the lower lip using a number 15 Bard Parker blade under local anesthesia. The tissue was excised carefully under an operating microscope to exclude underlying submucosal connective tissue or fat.

Limbal and conjunctival tissues were harvested after obtaining informed consent from patients undergoing limbal biopsy for cultivated limbal transplantation or from patients undergoing cataract surgery. The tissues were harvested under local anesthesia using the technique described earlier [9]. Eighteen limbal and ten conjunctival biopsies were used for the study. For biopsy, conjunctiva of the eye was incised 3 mm behind the limbus and dissection was continued toward limbus and then into the cornea for 1 mm using a number 15 Bard Parker blade. The conjunctiva was excised at the limbus just behind the pigmented line (palisades of Vogt), and the limbal tissue with 1 mm clear corneal tissue was excised. The tissues were carried to the laboratory in a human corneal epithelium (HCE) medium and processed in a similar way as described for oral mucosal biopsies.

**Table 1:** List of primers used in this study

Gene	Primers		Annealing Temperature	Product Size (bp)
Cytokeratin K3	Forward Reverse	GGCAGAGATCGAGGGTGTC GTCATCCTTCGCCTGCTGTAG	60 °C	145
Cytokeratin K12	Forward Reverse	ACATGAAGAAGAACCACGAGGATG TCTGCTCAGCGATGGTTTCA	60 °C	150
Cytokeratin K4	Forward Reverse	GCCATGATTGCCAGACAGCAGTGT GGGGGTGAGCAAGCTATGGTTG	58 °C	408
Cytokeratin K13	Forward Reverse	GATCCAGGGACTCATCAGCA AAGGCCTACGGACATCAGAA	58 °C	289
Cytokeratin K15	Forward Reverse	GGAGGTGGAAGCCGAAGTAT GAGAGGAGACCACCATCGCC	64 °C	193
Connexin43	Forward Reverse	CCTTCTTGCTGATCCAGTGGTAC ACCAAGGACACCACCAGCAT	60 °C	145
Pax-6	Forward Reverse	ATA ACC TGC CTA TGC AAC CC GGAACTTGAACTGGAACTGAC	55 °C	208
ΔNp63α	Forward Reverse	GGAAAACAATGCCCAGACTC ATGATGAACAGCCCAACCTC	60 °C	1389
ΔNp63β	Forward Reverse	GGAAAACAATGCCCAGACTC CAGACTTGCCAGATCCTGAC	60 °C	1376
ΔNp63γ	Forward Reverse	GGAAAACAATGCCCAGACTC GGGTACACTGATCGGTTTGG	60 °C	1168
p75	Forward Reverse	TGA GTG CTG CAA AGC CTG CAA TCTCATCCTGGTAGTAGCCGTAG	54 °C	230
GAPDH	Forward Reverse	GCCAAGGTCATCCATGACAAC GTCCACCACCCTGTTGCTGTA	55 °C	498

Primer sequence, annealing temperature, and size of amplicon in the RT-PCR analysis carried out in this study.

### Preparation of human amniotic membrane

The human amniotic membrane (HAM) was prepared from human term placenta obtained during Caesarian deliveries and processed at the Ramayamma International Eye Bank of L. V. Prasad Eye Institute as reported earlier [22]. Briefly, the placenta was washed repeatedly under sterile conditions with Ringer’s solution containing antibiotics. The amniotic membrane was peeled off from the chorion, rinsed using Ringer’s solution, and placed on nitrocellulose paper, keeping the epithelium side up. HAM was then stored at −70 °C in Dulbecco’s Modified eagles Medium (DMEM) in 50% glycerol. Prior to use, HAM was thawed at 37 °C for 30 min, peeled from the nitrocellulose membrane, and placed on a glass slide with the epithelium side up. HAM was deepithelialized using 0.25% Trypsin-EDTA at 37 °C for 30 min followed by mechanical scraping and washing with phosphate buffered saline (PBS).

### Cultivation of limbal, conjunctival, and oral mucosal epithelial cells

Tissue culture media, growth factors, and fine chemicals were obtained from Sigma-Aldrich (St. Louis, MO). The oral biopsy was washed three times with PBS containing antibiotics (penicillin, streptomycin, gentamicin, and amphotericin). The tissue was cut into small pieces using a sterile surgical blade (number 21) and placed on deepithelialized HAM. Tissue pieces were allowed to adhere to the deepithelialized HAM and then cultured in an HCE medium, which contained Minimal Essential Medium (MEM) and Ham’s F12 in a 1:2 ratio along with epidermal growth factor, insulin, penicillin, streptomycin, amphotericin, gentamicin, and 10% fetal calf serum for a period of three to four weeks in a humidified incubator at 37 °C with 5% CO_2._ The medium was changed every other day. Limbal and conjunctival biopsies were cultured in a similar manner.

### Hematoxylin-eosin staining

Two- to three-week-old cultures were fixed in 10% formalin and embedded in paraffin. Serial sections (five μm thick) of cultivated epithelia were generated. Sections were deparaffinized, rehydrated with distilled water, and stained with hematoxylin and eosin. Sections were observed under a light microscope.

### Periodic acid Schiff staining

Paraffin sections were deparaffinized, rehydrated, and then treated with periodic acid for 5 min. The sections were washed, treated with Schiff’s reagent for 15 min, and stained with hematoxylin.

### Transmission electron microscopy

Cells cultivated on HAM were fixed in 2.5% glutaraldehyde in PBS for 24 h and then post-fixed in 1% osmium tetroxide. Samples were dehydrated in a series of alcohol grades and embedded in Spurr’s resin. Ultrathin sections were cut using a Leica UltraCut UCT-GA-D/E-1/00 ultramicrotome (Leica, Wetzlar, Germany), stained with uranyl acetate, and counter-stained with 4% lead citrate. Sections were scanned in a transmission electron microscope (H-7500; Hitachi, Tokyo, Japan) at 80 kV.

### Reverse-transcription polymerase chain reaction

Total RNA was extracted from two- to three-week-old cultures using Trizol^TM^ (Invitrogen, Carlsbad, CA) according to the manufacturer’s protocol. The RNA was treated with DNase, and 4 µg of RNA was used for cDNA synthesis using Moloney Murine Leukemia Virus reverse transcriptase (MBI Fermentas, Vilnius, Lithuania). A polymerase chain reaction (PCR) was performed on this cDNA using the primers shown in Table 1, and the PCR products were separated on a 1.2% agarose gel.

### Immunohistochemistry

The anti-cytokeratin K3/K12 antibody, AE5 (used at 1:300), was obtained from Chemicon (Temecula, CA). The anti-p63 antibody 4A4 (used at 1:400) that recognizes all isoforms of p63 was obtained from Neomarkers (Fremont, CA). The anti-p75 antibody was obtained from Abcam (Cambridge, MA) and used at 1:200. Serial sections (five μm thick) of cultivated epithelia were subjected to immunohistochemistry. Briefly, the sections were deparaffinized, rehydrated, and blocked for endogenous peroxidase using 3% H_2_O_2_ in methanol. Antigen retrieval was done using a citrate buffer (pH 6.0) treatment in a microwave oven for 20 min, then allowing it to cool to room temperature. Nonspecific sites were blocked using 2.5% bovine serum albumin (BSA) after which the sections were incubated overnight at 4 °C with the primary antibody. Detection of the bound antibody was performed using Super Sensitive Non-Biotin HRP detection system (BioGenex, San Ramon, CA) according to the manufacturer’s instructions. Sections were counterstained with hematoxylin, mounted in a resinous mounting medium, and observed under a light microscope.

## Results

### Morphological characteristics of oral epithelial cells

**Figure 1:**
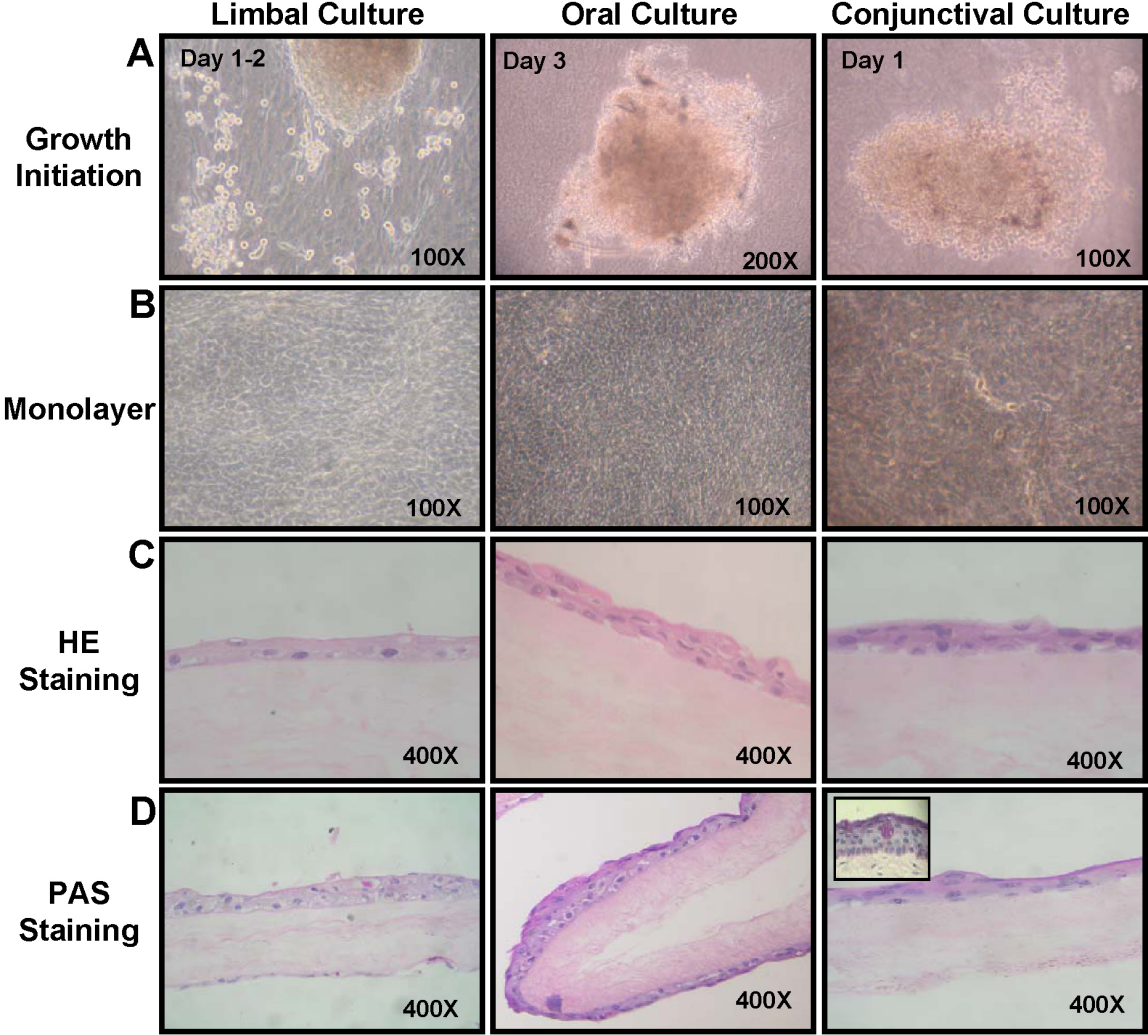
Growth initiation and morphological characteristics of limbal, oral, and conjunctival cultures. **A**: Growth initiation from the three explant cultures is shown. **B**: Confluent cultures of limbal, oral, and conjunctival cells are shown. **C**: Hematoxylin and eosin staining of sections of limbal, oral, and conjunctival cultures are illustrated. **D**: PAS staining of the three explant cultures is also shown. The inset in conjunctival culture shows goblet cells detected in native conjunctival tissue.

Oral, limbal, and conjunctival cells were cultured as explants on deepithelialized HAM using HCE medium. Compared to limbal and conjunctival cultures, cell growth initiation was slower in the oral cultures. Cell migration and growth initiated from oral explants within three to four days, which was later than that observed for limbal and conjunctival cultures (one to two days) as shown in Figure 1A. Healthy, confluent cultures of oral epithelial cells were obtained within three to four weeks (Figure 1B) as compared to 10–14 days in the case of limbal and conjunctival cultures. Cultured oral cells appeared to be slightly smaller in size compared to the cultured limbal or conjunctival cells. Even without airlifting, confluent oral epithelial cell cultures underwent stratification in places and formed two to three layers of cells as seen after hematoxylin-eosin (HE) staining (Figure 1C). No goblet cells were seen in these cultures by periodic acid Schiff (PAS) staining (Figure 1D).

**Figure 2:**
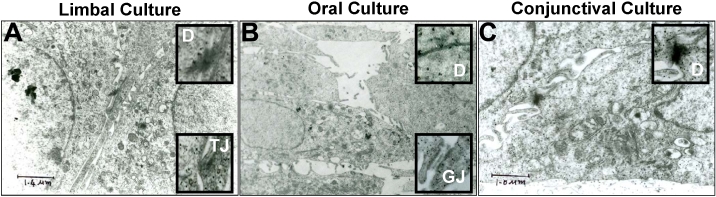
Ultrastructural studies of limbal, oral, and conjunctival cultures. **A**: The limbal epithelial cell is shown with the desmosomes (D) and tight junction (TJ) revealed in the inset. **B**: Oral epithelial cell is also shown with the desmosomes (D) and gap junction (GJ) revealed in the inset. **C**: Conjunctival epithelial cell is shown with just the desmosomes (D) demonstrated in the inset.

Electron microscopy was used to check the presence of cell-cell junctions and cell-basement membrane junctions. As shown in Figure 2B, cultured oral epithelial cells were able to form gap junctions and desmosomes with each other, which is similar to limbal and conjunctival cultures. However, hemidesmosomes, the junctions between cells and the amniotic membrane, were not clearly visible in any of these cultures under this resolution, although cells from all three cultures were in close apposition with the amniotic membrane (data not shown).

### Phenotypic characterization of oral epithelial cells

**Figure 3:**
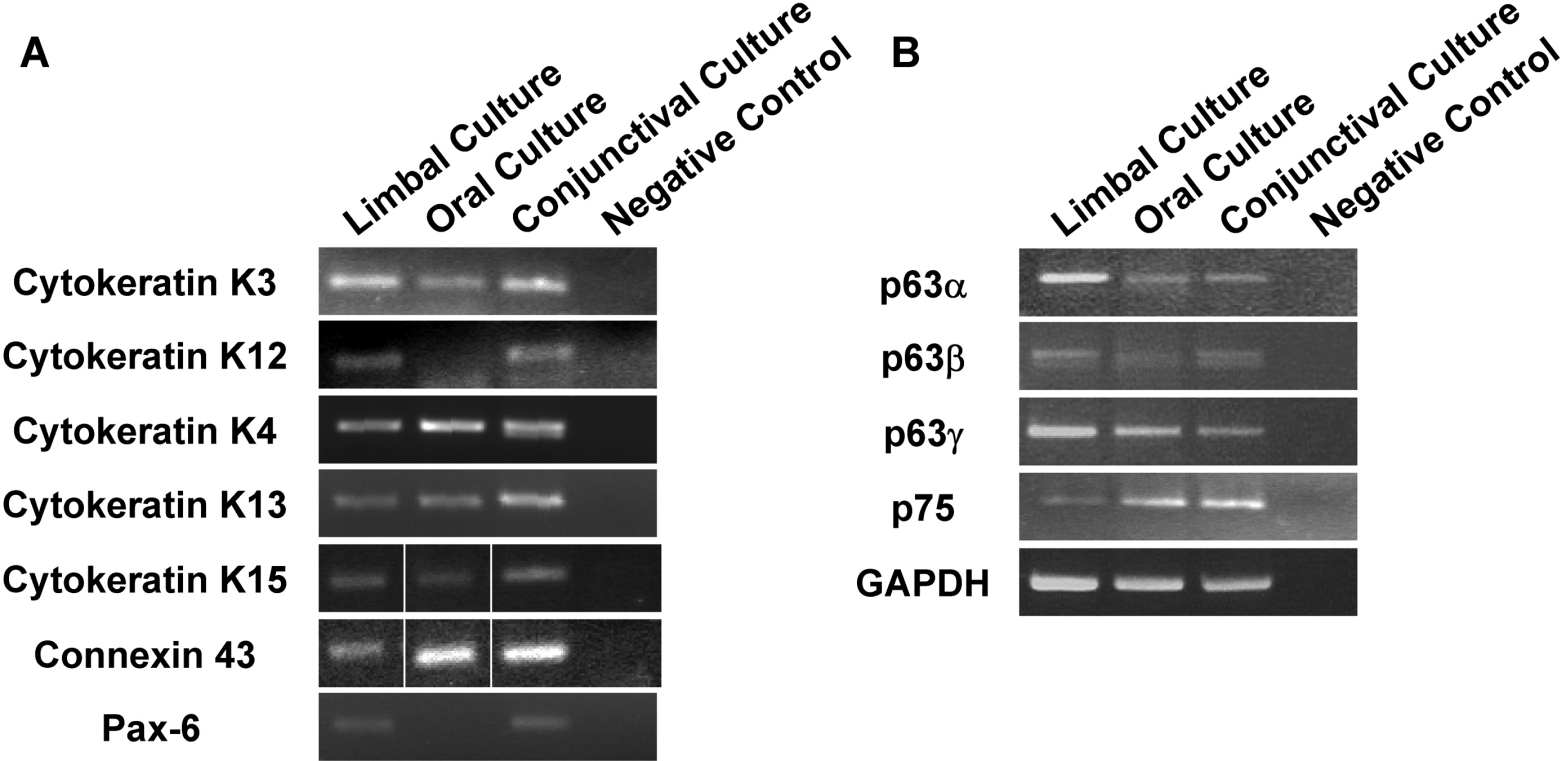
Expression of markers of epithelial stem cells and differentiation. RT-PCR analysis of expression of markers for epithelial differentiation (**A**) and stem cells (**B**) is shown. GAPDH was used as the normalizing control. The RT-PCR results of cytokeratin K5 and connexin 43 show a composite picture of PCR of cDNA from all three cells and the negative control that were performed in the same experiment. All experiments were performed twice with RNA isolated from a separate set of cultures.

Expression of different markers for stem cells as well as differentiated epithelial cells in cultured oral, limbal, and conjunctival cells was checked by reverse-transcription polymerase chain reaction (RT–PCR) analysis. cDNA was synthesized from RNA isolated from confluent cultures and was subjected to PCR using the primers shown in Table 1. Both limbal and oral epithelial cells expressed cytokeratin K3, the marker for differentiated corneal epithelial cells (Figure 3A). The oral epithelial cultures did not express cytokeratin K12, indicating that the cells retained the phenotype of oral epithelial cells. Interestingly, conjunctival cultures also expressed cytokeratin K3 and K12. The cells also expressed cytokeratin K4 and K13, markers of nonkeratinized stratified oral epithelia [23,24] as seen earlier in rabbit oral epithelial cultures [16]. These cytokeratins were also expressed by cultured limbal and conjunctival cultures. Cytokeratin K15, expressed in basal and suprabasal cells of the limbus [25], was also observed in all three cultured cells. Connexin 43, another marker for differentiated epithelial cells, was also expressed by these cells. Cultured oral cells did not express Pax-6, a marker for ocular tissues.

RT–PCR analysis was also performed to check for the presence of stem cell markers, namely, isoforms of p63, which is a stem cell-associated marker in all stratified epithelia, and of p75, a marker for stem cells in the oral epithelia [26]. Previous studies have shown that the limbal cultures express p63, specifically ΔN isoforms of p63 [27,28]. All three cultured cells expressed ΔNp63α, ΔNp63β, and ΔNp63γ (Figure 3B). No expression of transactivating isoforms of p63 was seen in any of the cultures (data not shown). Recently, p75 has been shown to be a marker for oral stem cells [26], and the expression of p75 has also been observed in the basal cells of the limbus, suggesting that it could be a marker for corneal progenitor cells [29,30]. As shown in Figure 3B, all three cell types expressed p75 as well.

**Figure 4:**
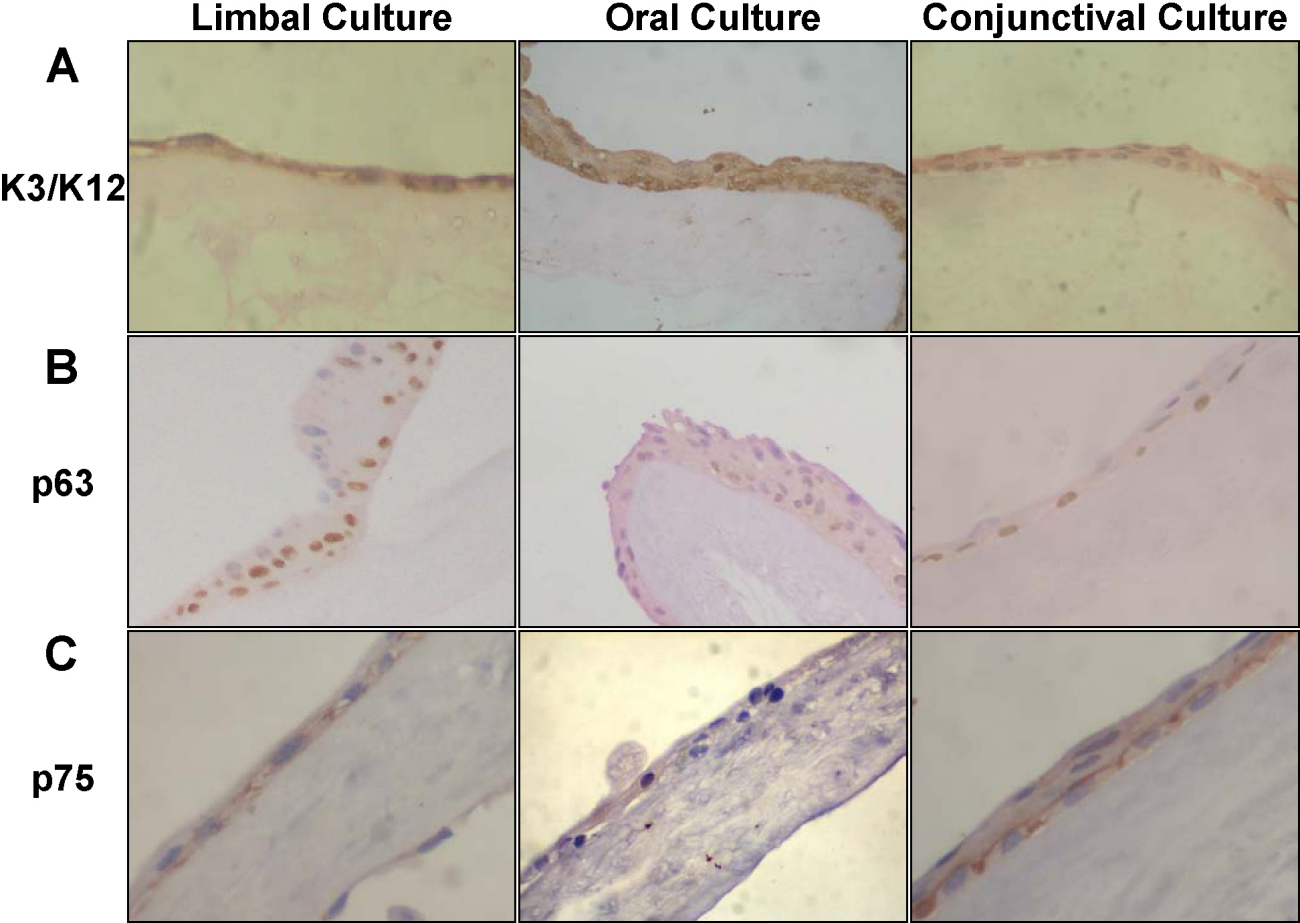
Expression of cytokeratin K3/K12, p63 and p75 in limbal, oral and conjunctival cultures. Immunohistochemistry was performed to detect the expression of cytokeratin K3/K12 (**A**), p63 (**B**), and p75 (**C**) in cultured limbal, oral, and conjunctival cells. The experiments were performed twice using sections from separate set of cultures and cells were observed at 400X magnification.

These results were further substantiated by immunohistochemistry of the cultured cells. Immunohistochemistry, using AE5 that recognizes both K3 and K12, showed that these cytokeratins were expressed in all three cultures. We believe that the signal seen in oral cultures is only due to cytokeratin K3 as we did not detect the expression of K12 by RT–PCR. Immunohistochemistry, using anti-p63 antibody, which recognizes all forms of p63, showed the expression of p63 isoforms in all three cultured cells (Figure 4). Expression of p75 was also checked by immunohistochemistry (Figure 4C). Only a very low expression of p75 was observed in the cultured oral cells in contrast to many of the cells from the cultured limbal and conjunctival cultures, which expressed p75.

## Discussion

We report here the successful cultivation of oral mucosal epithelial cells by using deepithelialized amniotic membrane without the use of feeder cells and the explant culture technique. The cultures were healthy, became confluent in three to four weeks, and underwent stratification. The cells formed junctions with each other. Phenotypic studies indicate that the cultures are a heterogeneous population of cells expressing markers of differentiated epithelial cells as well as stem cells. These oral epithelial cultures can now be used for ocular surface reconstruction in patients with bilateral LSCD. The advantage of the technique used here is that it precludes the use of feeder cells, which are of animal origin. Although feeder cell-free cultures of oral epithelial cells have been established using a temperature-responsive culture surface [21], such a technique has not been reported so far using the amniotic membrane. Our technique has overcome the need of feeder cells while retaining the advantages of the amniotic membrane. As these cells can also be cultured in the presence of an autologous serum (data not shown) as has been reported earlier [31], this technique reduces the risk of introducing xenobiotic agents into the patient.

We also compared the morphological and phenotypic characteristics of cultivated limbal, conjunctival, and oral epithelial cells. Our studies indicate that the three cultures express markers of stratified epithelia such as cytokeratin K3, K4, K13, K15, and connexin 43. The cultured oral cells do not express cytokeratin K12 or the eye-specific transcription factor, Pax-6. The cultured oral epithelial cells thus maintain their original phenotype as has been reported by other groups [16,20]. The absence of cytokeratin K12 may not interfere with ocular surface regeneration. Although cytokeratin K12 is required for the integrity of the corneal epithelium as suggested by knockout studies [32], it is not necessary for the integrity of the oral epithelium.

The oral cells expressed stem cell-associated markers such as all α, β, and γ isoforms of ΔN p63 as seen by RT-PCR analysis and by immunohistochemistry. Where cultures underwent stratification, the expression of p63 was observed in the basal layer. Expression of p75 was also seen by RT-PCR analysis. However, by immunohistochemistry, few cells expressing p75 were observed. It is possible that p75 is a more appropriate marker for the stem cells of the oral epithelium. As the number of stem cells in the culture may not be very high, a few cells expressing p75 were observed. p75 was also expressed in limbal and conjunctival cultures as has been reported earlier [30,33].

Interestingly, the conjunctival cells cultured under these conditions displayed several characteristics similar to cultured limbal cells. The cells were free of goblet cells as shown by PAS staining as well as electron microscopy, although the native tissue had them. They also showed the presence of cytokeratin K3 and K12 as seen by both RT–PCR and immunohistochemistry. These cytokeratins are the hallmark of corneal differentiation and are rarely reported in conjunctival cells. The presence of clusters of corneal progenitor cells in conjunctiva has been reported, and it is possible that the expression of K3 and K12 observed in this study could be due to the proliferation of corneal stem cells in conjunctiva [34]. It is also possible that these differences are due to the different niches that the culture conditions used here provide and thereby lead to changes in the expression profile. Since limbal deficiency leads to conjunctivalization of the ocular surface, whether cultured conjunctival cells can be used for ocular surface construction in cases of bilateral limbal deficiency remains to be seen. The efforts to reepithelialize the ocular surface using direct conjunctival transplants have shown that this surface undergoes vascularization [5]. A study performed in rats, using cultivated conjunctival epithelium, indicates that the corneal surface can be reconstructed using cultured conjunctival cells and the outcome of these grafts was better than an amniotic graft alone, but the study did not compare the results of ocular surface reconstruction using limbal grafts with that of conjunctival grafts [14]. Perhaps the option of using cultured conjunctival cells for ocular surface reconstruction can be explored further for cases where it is not feasible to use limbal or oral mucosal epithelial cells such as in cases of bilateral LSCD along with oral inflammation/infection.

Both conjunctival and oral epithelial cultures could lead to vascularization of the cornea that could in turn result in loss of vision. A long term follow-up of cultivated oral mucosal epithelial cell transplantation has indicated that while it leads to the formation of a stable ocular surface and improved visual acuity, it also leads to peripheral vascularization in the cornea [18]. A recent study indicates that the expression of FGF2 by oral epithelial cells could be responsible for the vascularization of the cornea after ocular surface reconstruction using oral epithelial cells [35]. This potential problem might perhaps be overcome by using anti-FGF2 antibodies.

To summarize, we have established cultures of oral mucosal epithelial cells on the human amniotic membrane without the use of feeder cells. The cultured cells are morphologically and phenotypically similar to cultured limbal cells. Using this technique, we have now initiated a clinical trial for ocular surface reconstruction in patients having bilateral limbal stem cell deficiency.
